# Dermatologic Toxicities of Targeted Therapy and Immunotherapy in Head and Neck Cancers

**DOI:** 10.3389/fonc.2021.605941

**Published:** 2021-05-27

**Authors:** Maria L. Espinosa, Chelsea Abad, Yaira Kurtzman, Farah R. Abdulla

**Affiliations:** ^1^ Department of Dermatology, University of Chicago Pritzker School of Medicine, Chicago, IL, United States; ^2^ Department of Dermatology, City of Hope Los Angeles, Duarte, CA, United States

**Keywords:** immunotherapy, dermatologic toxicity, adverse event, head and neck cancer, cetuximab, pembrolizumab, nivolumab, trastuzumab

## Abstract

Treatment of head and neck cancers requires multidisciplinary collaboration to reduce morbidity and mortality associated with the tumor burden, as well as to preserve function of organs and structures. With the use of various new targeted therapies come new adverse events including dermatologic toxicities, which may consist of xerosis, nail and hair changes, morbilliform or papulopustular rashes, to more severe eruptions such as Stevens–Johnson syndrome. We describe the dermatologic toxicities and corresponding grades of severity and associated pathophysiology resulting from seven therapeutics used to treat head and neck cancers: cetuximab, trastuzumab, pembrolizumab, nivolumab, lentatinib, larotrectinib, and entrectinib. Being familiar with these dermatologic toxicities allows clinicians to provide comprehensive counseling for patients, encourage preventative measures, and to know when it is appropriate to hold therapy or permanently stop treatment.

## Introduction

Head and neck (H&N) cancers are those that primarily affect the lip, oral cavity, pharynx, larynx, and paranasal sinuses (1). The incidence of these cancers is rising, with over 60,000 new cases reported each year in the United States. Major risk factors for developing H&N cancers include heavy tobacco and alcohol use, as well as human papillomavirus (HPV) infection (2). Treatment requires multidisciplinary collaboration to reduce morbidity and mortality associated with the tumor burden, as well as to preserve function of organs and structures. With the use of various new targeted therapies come new adverse events including dermatologic toxicities which may range from a limited morbiliform rash to diffuse bullous eruptions and more severe manifestations. These dermatologic toxicities can greatly impact a patient’s quality of life so clinicians must be familiar with these toxicities, know how to manage them, and recognize when it is necessary to hold or permanently stop the targeted therapies. In this review we aim to describe the dermatologic toxicities and corresponding treatments associated with the following H&N cancer therapeutics: cetuximab, trastuzumab, pembrolizumab, nivolumab, lentatinib, larotrectinib, and entrectinib. We will cover common skin reaction patterns resulting from targeted therapies and immunotherapies and then will review each of these seven therapeutics independently.

## Common Drug Induced Skin Reaction Patterns

Common dermatologic toxicities that arise in the treatment of head and neck cancers include papulopustular eruptions, paronychia and other nail changes, mucositis, xerosis, and bullous eruptions. Descriptions, corresponding inciting drugs, and severity staging are found in **Table 1**. Severity staging is defined by the Common Terminology Criteria for Adverse event guidelines (22). Papulopustular reactions are pruritic eruptions consisting of papules and pustules along the head, neck, trunk, and upper extremities. Historically, these papulopustular eruptions have been closely associated with EGFR/HER1 inhibition (15). These lesions occur in a seborrheic dermatitis-like pattern on the scalp, face, and chest and are often pruritic which is not common in other acneiform eruptions (15). It is important to note that papulopustular eruptions in these patients are not acne; the skin is devoid of comedones with this rash (23). Oral mucositis is a painful inflammation of the mucosa of the oral cavity which may decrease a patient’s quality of life by making it difficult to eat and speak. Xerosis is more widespread and can even affect vaginal and perineal tissues. Xerotic skin facilitates eczematous dermatitis, and many patients experience eczematous patches and plaques on palmoplantar surfaces that can lead to painful fissuring of fingertips (15). Palmar-plantar erythrodysesthesia syndrome (PPES), or hand foot syndrome, is frequently associated with oncologic treatments and is comprised of dysesthesia, erythema, edema, and possible desquamation and blisters along the fingers and toes (24).

**Table 1:** Dermatologic skin reaction patterns resulting from targeted therapies and immunotherapies for head and neck cancers.

	Papulopustular/acneiform eruption	Morbilliform eruptions	Palmar plantar erythrodysesthesia syndrome (PPES), also known as hand foot syndrome		Oral mucositis	Paronychia	Bullous pemphigoid
**Description**	Papules and pustules along the head, neck, trunk, and upper extremities with or without pruritus and tenderness.	Macules and papules, typically along upper torso, spreading centripetally, with or without pruritus.	Erythema, edema, and possible desquamation and blisters along the fingers and toes with or without pain.		Inflammation or ulceration of the mucosa of the oral cavity which may decrease a patient’s quality of life by making it difficult to eat and speak.	Inflammation along the soft tissues of the nail with variable superinfection.	Pruritic tense vesiculobullous eruptions along torso and extremities, frequently with mucosal involvement.
**Severity scoring according to CTCAE guidelines^a^**	Grade 1: papules and/or pustules covering <10% BSA (with or without pruritus/tenderness) Grade 2: papules and/or pustules covering 10–30% BSA, with or without pruritus/tenderness, associated psychosocial impact III: papules and or pustules covering >30% BSA with moderate to severe symptoms, associated with superinfection Grade 4: life threatening consequences, urgent intervention needed Grade 5: death related to adverse event	Grade 1: macules/papules covering <10% BSA), with or without symptoms Grade 2: 10–30% BSA with or without symptoms; limiting instrumental ADL Grade 3: macules and papules covering >30% BSA with moderate to severe symptoms; limiting self-care ADL	Grade 1: minimal skin changes or dermatitis (erythema, edema, hyperkeratosis), non-painful Grade 2: Skin changes (Peeling, blisters, fissures, edema, or hyperkeratosis) with pain, limiting instrumental ADL Grade 3: severe skin changes with pain, limiting self-care ADL		Grade 1: asymptomatic or mild symptoms. Grade 2: moderate pain or ulcer, modified diet indicated Grade 3: severe pain; interfering with oral intake Grade 4: life-threatening consequences Grade 5: death	Grade 1: nail fold edema or erythema, cuticle disruption Grade 2: local and oral intervention indicated; pain along nail fold with edema or erythema, associated with discharge or nail plate separation Grade 3: limited self-care ADL; operative intervention and IV antibiotics indicated	Grade I: asymptomatic blisters covering <10% BSA Grade 2: bullous dermatitis (painful blisters covering >30% BSA) Grade 3: blisters covering >30% BSA, limited self-care, fluid and electrolyte abnormalities Grade 4: life threatening consequences, urgent intervention needed Grade 5: death related to adverse event
**Treatments**	Grade 1: topical antibiotic agents (erythromycin/clindamycin) +/− topical corticosteroids if there is an inflammatory component. Grade 2: oral antibiotics Grade 3 or higher: Stop anti-EGFR therapy and provide oral corticosteroids an antibiotics until inflammation was decreased (3–6) -Isotretinoin or acitretin for EGFR-inhibitor induced papulopustular eruptions (7, 8)	Grade 1: continue the immunotherapy and provide supportive care with topical emollients, oral antihistamines, and topical steroids to affected areas. Grade 2 toxicity: hold immunotherapy, provide supportive care, and prescribe prednisone 0.5–1 mg/kg/day. Grades 3–4: stop immunotherapy, apply high potency topical steroids to affected areas, start prednisone (up to 2 mg/kg/day), and providers may consider inpatient care (9). Notes: Once Grade 2–3 eruptions improve to Grade 1, immunotherapy may be re-started. However, patients that experience a Grade 4 toxicity should discontinue the inciting drug permanently (10, 11).	Grade 1: application of a moisturizer is recommended. Grade 2: topical corticosteroids or urea creams. Grade 3: consider stopping drug until symptoms resolve to Grade ≤1 (12).		Prophylaxis: good oral hygiene, avoidance of mint-flavored toothpaste or alcohol containing mouthwash. -Topical corticosteroids, topical lidocaine, or triamcinolone paste (6, 12) for painful ulcerations. -Dexamethasone mouthwash (13).	Prevention with properly trimmed nails, avoidance of ill-fitting shoes, and diluted bleach soaks (14). For acute inflammation with serosanguinous drainage: warm soaks, a high potency topical corticosteroid and antimicrobial, such as betamethasone dipropionate 0.05% ointment and gentamycin 0.05% cream (15). Incision and drainage if abscess develops; consider systemic antibiotics if drainage not fully successful or overt cellulitis (16) Biotin supplementation has proven in some studies to be efficacious for enhancing nail plate strength after becoming brittle from EGFR inhibitor treatment (17).	Grade 1: hold immunotherapy and start high potency topical steroids to the affected areas. Grade 2: hold immunotherapy and prescribe prednisone/methylprednisolone 0.5–1 mg/kg/day. Grade 3: permanently stop the immunotherapy, prescribe prednisone/methylprednisolone 1–2 mg/kg/day, admit patient, consider ophthalmology and urology consults if involvement of the eyes or genitals (9). If needed, steroid-sparing agents to consider include azathioprine, mycophenolate mofetil, methotrexate, tetracycline antibiotics, dapsone, or niacinamide (18). Recalcitrant BP: consider rituximab (19), omalizumab (20), or dupilumab (21).
**Inciting head and neck cancer targeted therapies and immunotherapy**	Trastuzumab, Afatinib	Pembrolizumab, nivolumab		Trastuzumab; lenvatinib	Lenvatinib	Cetuximab	Pembrolizumab, nivolumab

Staging is based on the Common Terminology Criteria for Adverse events guidelines.

ADL, activities of daily living; BSA, body surface area, BP, bullous pemphigoid.

## Targeted Therapy and Immunotherapy

### Cetuximab

Cetuximab is an epidermal growth factor (EGFR) inhibitor approved for the treatment of H&N cancers. Over half of patients with squamous cell carcinoma (SCC) will require a combination therapy due to advanced disease at diagnosis (1). Currently, the preferred standard of care is high-dose cisplatin with concurrent radiotherapy (1). For patients that are not candidates for cisplatin therapy, cetuximab combined with radiotherapy is the preferred regimen (1). In cases of non-pharyngeal SCCs that are recurrent, metastatic, or unresectable, the recommended treatment is a combination of cisplatin, cetuximab, and 5-fluorouracil. Patients with this specific H&N cancer that are deemed medically unfit for the chemotherapy agents in this combination therapy may receive treatment with single agent cetuximab (1). To date, cetuximab combined with radiotherapy is shown to be superior to radiotherapy use alone in stages III–IVB SCCHN. There are no randomized controlled trials that demonstrate with statistical significance that cetuximab and radiotherapy combination is superior to chemotherapy agents, specifically cisplatin and radiotherapy (25). Yet, it is recommended that treatment plans are patient-specific and developed in collaboration with other specialties, like radiation oncology, surgery, and supportive medicine to administer medication safely with respect to prognosis, feasibility, and patient characteristics (1).

Squamous cell histology dominates the cellular lineage in H&N cancers, and epidermal growth factor (EGFR) is almost always expressed in squamous cell carcinoma of the H&N (SCCHN) (2). EGFRs represent a diverse set of ligands of the receptor tyrosine kinases (RTKs) that transduce extracellular signals through intracellular activation to exact the specific functions of growth factors (26). Overexpression of EGFR leads to gene amplification, aberrant cellular proliferation, and is one of the mechanisms identified in human malignancies which have sparked massive effort in the development of targeted therapies for anti-cancer properties. Cetuximab is one such therapy aimed at inhibiting EGFR function *via* competitive binding of the receptor’s extracellular domain. It is a chimeric monoclonal antibody of immunoglobulin G1 class and exhibits more affinity for EGFR than endogenous ligands, making cetuximab effective at binding to the target (2).

Cetuximab has been reported to cause a variety of skin reactions (15). The human skin, specifically epidermal keratinocytes and pilosebaceous units, are replete with EGFR ligands (27). Hence, inhibition of EGFRs by both small-molecule EGFR inhibitors and anti-EGFR antibodies exerts inflammatory and toxic effects on the skin. Blocking the domain function of EGFR leads to inhibition of DNA synthesis and transcriptive functions, which in turn increases the terminal keratinization (17). The resulting thinned epidermis impairs the protective function of the skin (17). EGFR or ERK inhibition also leads to aggravation of the skin inflammatory response with upregulated chemokine expression as evidenced by tissue samples displaying dermal infiltration by T cells and macrophages (27). These repeatedly discovered inflammatory patterns lead to skin toxicities related to alteration of EGFR and not off-target effects of inhibitors (23).

Adverse cutaneous reactions with use of cetuximab for the treatment of SCCHN occur in greater than 80% of patients (15). Hair and nails can also be affected in about 10–20% of patients. Common reactions related to skin, hair, and nails are described as papulopustular rash, pruritus, xerosis, paronychia, hair abnormalities, and mucositis (15). Papulopustular eruptions are the most common cutaneous reaction with cetuximab use, affecting 60–80% of patients (15). Most patients will have a mild to moderate reaction, with less than 20% of patients experiencing a severe reaction. Typically, these erythematous, papular, and pustular lesions manifest within one to three weeks of starting cetuximab, often peaking and worsening around week five.

Xerosis is present in about 35% of patients treated with cetuximab and causes eczematous patches and plaques on palmoplantar surfaces that can lead to painful fissuring of fingertips (15). One study found that within six weeks of initiation of treatment with an EGFR inhibitor, patients developed exsiccation and exfoliation, leading to complaints of pruritus (28). Specifically, the study found that horny layer moisture content of the stratum corneum decreased significantly and seemed to be more exaggerated in the upper extremities (28). Nail and hair changes are rare adverse events and typically develop after several weeks up to several months of cetuximab use (15). Nail toxicity can encompass a variety of physical changes, such as pitting, discoloration, onycholysis and lead to the development of acute paronychia (**Table 1**). Hair changes include the hair becoming curly or wavy, brittle or fine texture, and alopecia of the scalp or beard (23). Eyelashes can also grow out long and rigid, causing pain and keratitis if growing inward (23). There are few preventative methods that prove to be efficacious at avoiding these hair and nail changes, but it is recommended that patients trim eyelashes regularly and perform daily antiseptic soaks for nails. In general, there is a lack of rigorous clinical trials aimed at assessing and identifying prophylactic measures to avoid the development of cutaneous side effects of EGFR inhibitors.

### Trastuzumab

Trastuzumab is approved for treatment of adjuvant breast cancer and metastatic breast and gastric cancers that are positive for human epidermal growth factor receptor 2 (HER2). Trastuzumab therapy has also been explored to treat various other malignancies where HER2 is overexpressed such as cutaneous and head and neck squamous cell carcinomas (SCCs) as well as cervical adenocarcinomas (29, 30). Trastuzumab is a monoclonal antibody engineered to target HER2 receptors. HER2 receptors are traditionally found in low levels in the epithelial cells of a variety of tissues (31). However, HER2 in HER2+ breast, GI, and various other cancers, have been shown to be amplified making HER2 an attractive anti-cancer target (29, 32, 33). HER2 plays a significant role in cell proliferation signaling pathways, and therefore alterations in HER2 expression have been linked to cancer’s hallmark trait of relentless and uncontrolled growth (31, 34).

Although the precise mechanism of action of trastuzumab is not fully understood, it is believed to block intracellular signaling pathways. When blocked, apoptosis and a slowing of cell proliferation are observed. This blocking prevents the activation of HER2 by its proper activators, promotes antibody-dependent cellular cytotoxicity through natural killer cells, and helps prevent HER2 shedding (35–37).

HER1 receptors are expressed in the skin in keratinocytes in the basal layer. Thus, HER1 inhibitors disrupt these cells’ development resulting in stratum corneum such as follicular infundibulum. In addition, the inhibitor promotes chemokine expression leading to apoptosis of keratinocytes (38). These numerous changes result in hyperkeratosis, follicular plugging, and inflammation which then manifest as a papulopustular rash which is a common and well-established side effect of HER1/EGFR inhibitor treatments as it is reported to occur at some point during therapy in 60–90% of treated patients (38). HER2 has been also detected in keratinocytes in the upper spinous layers and both HER1 and HER2 heterodimers are found in keratinocytes albeit at very low levels. Thus, it is hypothesized that trastuzumab by inhibiting HER2 in the skin causes papulopustular eruptions *via* HER2 homodimer inhibition or HER1–HER2 heterodimer inhibition.

Rare cutaneous adverse reactions to trastuzumab have been noted to occur. In one woman who received monotherapy with trastuzumab, tufted hair folliculitis was observed (39). Also, albeit uncommon, rash associated with a serious infusion reaction was noted in less than 0.3% of patients. Mild to moderate infusion reactions were found to be more common with the combination of trastuzumab and chemotherapy compared with chemotherapy alone (40). Another uncommon adverse event is carotenoderma, also referred to as carotenosis cutis, and aurantiasis cutis refers to the manifestation of yellow-orange skin coloration resulting from carotenemia (41). There have also been reported cases of trastuzumab induced dermatomyositis, a complement-mediated idiopathic inflammatory myopathy manifested by skin changes and proximal muscle weakness (42).

### Pembrolizumab and Nivolumab

Pembrolizumb is a humanized IgG4 monoclonal antibody serving as an immune checkpoint inhibitor by targeting the programmed cell death 1 (PD-1) receptor on activated T cells. When PD-1 is engaged by a ligand, PD-1 inhibits the kinase signaling pathways that usually leads to T-cell activation through phosphatase activity (43). In a phase 3 open label trial (KEYNOTE-048) comparing pembrolizumab alone, pembrolizumab with platinum and 5-fluorouracil, and EXTREME therapy (cetuximab, platinum, and 5-fluorouracil), pembrolizumab was determined to be an appropriate first line treatment for recurrent or metastatic head and neck squamous cell carcinoma (HNSCC) with platinum and 5-fluorouracil or as monotherapy for patients with PD-L1 positive tumors (44, 45). Nivolumab is another humanized IgG4 monoclonal antibody which targets the PD-1 receptor. In 2016, two months after pembrolizumab was approved for the treatment of HNSCC, the Food and Drug administration (FDA) approved nivolumab for treating platinum refractory HNSCC after the results from the CheckMate 141 phase III clinical trial showed a median overall survival of 7.5 months in the nivolumab group *versus* 5.1 months in the group with standard single-agent systemic therapy (methotrexate, docetaxel, or cetuximab) (46, 47).

Cutaneous reactions are common with anti-PD-1 therapy with about half of all patients developing some kind of cutaneous toxicity (48). The most common dermatologic adverse events (dAEs) that arise after treatment with pembrolizumab and nivolumab include pruritus, morbilliform eruptions, and lichenoid eruptions (10, 49). Less common dAEs include vitiligo, bullous pemphigoid, psoriasis; even more rare yet highly morbid dAEs include Stevens–Johnsons syndrome (SJS), Toxic Epidermal Necrolysis (TEN), and drug reaction with eosinophilia and systemic symptoms (DRESS) (49).

Pruritic morbilliform eruptions are non-specific findings seen as a result of many medications, and their diagnosis relies on a thorough history of all medications and timing of the resulting skin eruptions to identify the culprit drug. PD-1 inhibitor induced morbilliform eruptions typically present three to six weeks after initial dose with erythematous macules and papules coalescing into plaques, primarily over the trunk (10, 50). Lichenoid drug eruptions present typically on the torso as red to violaceous papules and plaques or flat topped papules and erosions along the oral mucosa about 12 weeks after initial dose, with a range of 1–266 days (51). Incidence rates for morbilliform and lichenoid eruptions after PD-1 therapy are very similar and have been seen to arise in about one fifth of patients who receive anti-PD-1 therapy (51, 52). Lichenoid drug eruptions are diagnosed based on exam findings, histology that reveals a dense, band-like lymphocytic infiltrate in the dermis, and a thorough history of medication (11, 53).

Vitiligo is an autoimmune skin disorder presenting with localized or generalized hypopigmented patches from the loss of melanocytes in the epidermis. In 2020, analysis of the World Health Organization pharmacovigilance database showed an association between vitiligo and pembrolizumab with a reporting odds ratio (ROR) of 116.9 (95%CI 94.8, 144.3) and between vitiligo and nivolumab (ROR 22.6, 95% CI 15.8, 32.4) (54). The occurrence of vitiligo typically occurs several months after initiation of PD-1 inhibition (50). The development of vitiligo in melanoma patients treated with PD-1 inhibition is well documented, but only few case reports have shown this association in patients with solid tumors treated with PD-1 inhibition (55, 56). Furthermore, the first case of pembrolizumab induced vitiligo in a patient being treated for HNSCC was reported in 2019. The author describes a 32-year-old man with stage IVA T2N2M0 squamous cell cancer of the tonsil and achieved complete remission with docetaxel, carboplatin, 5FU, and radiation but relapsed two years later and was treated with pembrolizumab after IHC of the biopsy demonstrated 90% PD-L1 expression (55). Five months after stopping pembrolizumab, he developed a few hypopigmented patches on his face that were biopsy proven to be consistent with vitiligo. The mechanism behind PD-1 inhibitor associated vitiligo is likely due to aberrant recognition of antigens in the dermis and epidermis by reactivated CD4+/CD8+ T cells, thereby leading to a potent inflammatory process (55). In most cases, the PD-1 inhibitor was continued despite the occurrence of vitiligo. Treatment recommendations for cosmetic reasons includes topical corticosteroids, strict sun protection, and phototherapy if disease extent is diffuse (57–59).

Bullous pemphigoid (BP) typically arises weeks to months after initiation of anti-PD-1 and anti-PD-LI therapy (49). PD-1 inhibitor induced BP is a rare but well established association with an estimated incidence of 1–2% according to two retrospective reviews at single institutions (48, 60–62). An analysis of the FDA Adverse Event Reporting System found a proportional reporting ratio of 5.87 for nivolumab and 6.36 for pembrolizumab used across many cancers, showing that this association is more common with pembrolizumab (63). Unlike the non-specific morbilliform and lichenoid drug eruptions, the diagnosis of BP can easily be made with direct and indirect immunofluorescence assays, quantification of circulating autoantibodies against BP180 and/or BP230, and physical exam findings. The pathophysiology of the development of BP may be due to the recognition of common antigens BP180 and BP230 shared between cutaneous basement membrane and tumor cells (64). Additionally, PD-1 inhibition can activate B cells and inhibit immunosuppressive B regulatory cells, thereby unmasking BP (65). Patients that develop BP after PD-1 inhibition may already have antibodies against BP180 and BP230, which is why pembrolizumab and nivolumab may unmask BP by further activating these B cells and unleashing the existing antibodies. Unlike other types of drug-induced BP, PD-1 inhibitor induced BP may even persist up to one year after cessation of immunotherapy likely due to sustained immune activation associated with anti-PD-1 therapy and may require maintenance therapy.

Psoriasis clinically presents with well-demarcated, scaly erythematous patches and plaques on the trunk and extremities, typically developing days to months after initiation of PD-1 inhibitors and has been seen in patients with and without a previous history of psoriasis (66). Exacerbation of psoriasis in patients with an established history of psoriasis tend to flare within a few days of immunotherapy, and *de novo* tends to appear months after initiation (67). The prevalence and incidence rates of psoriasis with anti-PD1 inhibitors are lacking but a literature review in 2018 revealed 35 reported cases, and it is clear that *de novo* is less common than flaring of established disease (48, 68). The pathogenesis of PD-1 inhibition induced psoriasis is due to the upregulation of pro-inflammatory Th-1/Th-17 pathways with elevated levels of interferon-gamma, tumor necrosis factor-alpha, and interleukins 2,6,17 (69). Since psoriasis is an autoimmune disease mediated by Th17, the upregulation of Th17 as a result of PD-1 inhibition is a likely culprit behind this dAE (68). Generally, patients with limited disease can tolerate continued immunotherapy (with prolonged intervals if needed) along with standard treatment for psoriasis including topical steroids, and topical vitamin D analogs (68, 70, 71). If psoriasis is recalcitrant or is affecting the patient’s quality of life, providers may consider cessation of immunotherapy and starting oral prednisone, acitretin, or phototherapy (67, 72).

Less common cutaneous toxicities include granulomatous reactions, erythema multiforme, SJS/TEN, and DRESS (59). SJS/TEN may present initially with a non-specific morbilliform eruption, later developing targetoid lesions, mucosal ulcerations and full thickness epidermal sloughing. This dAE can manifest weeks to months after the initial dose of a PD-1 inhibitor (49). Morbidity is high, and case reports have shown that stopping the medication does not greatly ameliorate symptoms. Treatment includes immediate cessation of inciting drug, close monitoring, and interventions such as prednisone 1–2 mg/kg/day, intravenous immunoglobulin, and cyclosporine (9, 73). A randomized controlled trial comparing the use of an etanercept and corticosteroids for cytotoxic T-lymphocyte mediated severe cutaneous adverse reactions showed decreased mortality and shorter skin healing time in the etanercept group (74).

In sum, PD-1 inhibitors cause a wide-range of dermatologic toxicities. Interestingly, some of these dAEs such as spongiotic dermatitis, vilitigo, and bullous pemphigoid arising after treatment with pembrolizumab have been associated with improved tumor response and survival outcomes, albeit in various cancers including melanoma, lung cancer, merkel cell carcinoma, and non-melanoma skin cancer (75, 76). Therefore, the presence of these toxicities may be a sign that the drug is working against the cancer as well, and providers may reassure patients by sharing this association and managing their symptoms as mentioned above.

### Afatinib

Afatinib is an orally administered irreversible tyrosine kinase inhibitor which halts adenosine triphosphate (ATP) binding to the intracellular domain of the epidermal growth factor (EGFR) receptor and blocks downstream signaling (77, 78). Afatinib has been found to be a promising therapeutic for the treatment of HNSCC since >80% of patients with HNSCC overexpress EGFR (79, 80). In a randomized phase III trial, 322 patients with recurrent or metastatic HNSCC were randomized to receive afatinib, and 161 patients received methotrexate. The group that received afatinib had longer progression free survival (2.9 *vs*. 1.7 months in the methotrexate group), improved quality of life, and was overall well tolerated. Notably, 215 (67%) of patients that received afatinib had grade 3 or higher adverse events, and the most common toxicities included rash and diarrhea. The term rash in this study encompassed a variety of conditions including but not limited to acne, dermatitis, dermatitis acneiform, erythema, folliculitis, and morbilliform rash. Furthermore, analysis of seven phase II/III studies using afatinib at a starting dose of 50 mg daily for 998 patients with various solid tumors found that 82% of patients experienced rash/acne (81). These morbilliform or acneiform eruptions are non-specific and make diagnosis and treatment difficult since oncology patients are frequently on various concomitant drugs that may be the culprit. Therefore, it is important to educate patients about this potential side effect so that it may be recognized and managed early.

Specifically the dermatologic toxicities seen with afatinib most commonly consist of a papulopustular rash, which typically arises two weeks after the initiation of therapy (82). Other dermatologic toxicities include paronychia, xerosis, pruritus, and cheilitis (3, 83). The pathophysiology of EGFRI-associated dermatologic toxicities is likely multidimensional. First, EGFR is essential for normal skin development since it is present on epidermal keratinocytes, sebaceous glands, and on the epithelium of hair follicles so EGFR inhibition leads to disruption in proliferation (84). Secondly, tyrosine kinase inhibitors may recruit additional inflammatory cells *via* secretion of chemokines that cause leukocyte chemotaxis and infiltration of follicles, leading to inflammation (4, 38).

### Lenvatinib

Lenvatinib is an oral tyrosine kinase inhibitor that blocks various receptors including vascular endothelial growth factor (VEGF) receptors 1–3, fibroblast growth factor (FGF) receptors 1–4, platelet derived growth factor (PDGF) receptor-*α*, RET, and KIT proto-oncogenes (85, 86). This drug is FDA approved for radioactive-iodine refractory thyroid cancers after results from a phase 3 multi-center study randomized patients to lenvatinib or placebo and found that those that received lenvatinib had significantly improved progression free survival (18.3 *versus* 3.6 months in placebo group) (87). However, 97% of the 261 patients in the lenvatinib arm experienced an adverse event of any grade, with 76% of patients experiencing Grade ≥3 adverse events compared to 10% in the placebo arm. Dermatologic toxicities resulting from lenvatinib include stomatitis in 20–36% of patients in clinical trials (87, 88), PPES in 32–75%, more commonly in patients of Japanese background (87, 89, 90), rash in 16% (87), and alopecia in 11% (87). These toxicities are a known class effect of VEGF inhibitors.

### Larotrectinib

Larotrectinib is approved by the FDA in November 2018 for the treatment of adult and pediatric patients with solid tumors that have a neuotrophic receptor tyrosine kinase (NTRK) gene fusion without known acquired resistance mutation, metastatic disease. Or in cases when surgical resection may result in severe morbidity (91). NTRK genes (NTRK1, NTRK2, NTRK3) code for tropomyosin receptor kinase (TRK) proteins (TRKA, TRKB, TRKC). The TRK proteins are mainly expressed on neural cells and later may fuse with other proteins, thereby leading to constitutively active downstream signaling (92). Larotrectinib blocks this fusion protein and prevents the downstream signaling, effectively blocking tumor progression in cancers with this fusion protein. Approval was based on data from three multicenter, open-label, single-arm clinical trials: LOXO-TRK-14001 (NCT02122913), SCOUT (NCT02637687), and NAVIGATE (NCT02576431) (92). Patients had various solid tumor types including 12 with salivary gland tumors and five with thyroid tumors. Analysis of the first 55 patients enrolled showed a 75% overall response rate by independent review (92). NTRK gene fusion mutations have been identified in 2.4–25.9% of thyroid cancer (93, 94) and only in less than 1% of head and neck squamous cell carcinoma (94). Therefore, larotrectinib is not the drug of choice for most head and neck cancers since not all tumors will have this targetable fusion protein.

Dermatologic toxicities associated with larotrectinib and other NRTK blockers are not well documented. The most common adverse events associated with larotrectinib in the trials that led to FDA approval include liver transaminsase elevations, anemia, fatigue, nausea, dizziness, and diarrhea (92, 95). The authors only reported adverse events that occurred in at least 15% of patients, so it is possible that cutaneous toxicities arose but were not reported since they were very rare. Based on the mechanism of action of larotrectinib blocking tyrosine kinase downstream signaling, it is possible that it may cause rash, pruritus, and painful skin-dermatologic side effects with entrectinib, another drug that works by inhibiting TRK (96). There are several open clinical trials assessing the long-term efficacy and tolerability of larotrectinib.

### Entrectinib

Entrectinib is an orally administered inhibitor of TRKA, TRKB, TRKC, ROS1, and ALK with the ability of crossing the blood–brain barrier (97). It was approved by the FDA in August 2019 after review of the findings of three ongoing, phase 1 or 2 clinical trials (ALKA-372-001, STARTRK-1, and STARTRK-2) (96). An analysis of these three pivotal trials included 54 patients with advanced or metastatic solid tumors with any NRTK gene fusion (seven had mammary analog secretory carcinoma, and five had thyroid cancer) and found efficacy (96). In the overall safety evaluable population (n = 355) across all three studies which included patients of any tumor type and gene rearrangement, dermatologic toxicities included rash (6%), pain of the skin (4%), and pruritus (5%) primarily of grades 1–2 (96). The pathophysiology of these cutaneous toxicities may be from the inhibition of TRKA receptors on human keratinocytes, thereby inhibiting phosphorylation and leading to reduced keratinocyte proliferation (98, 99). These toxicities can be treated similar to the pruritus and morbiliform eruptions seen with other immunotherapy and molecularly targeted therapies, and entrectinib can be safely continued (96).

## Treatments for the Dermatologic Toxicities Associated With Targeted Therapy and Immunotherapy for Head and Neck Cancers

Treatments for the common skin reaction patterns are described in **Table 1**. In general, papulopustular and morbilliform eruptions are the most common reaction patterns incited by drugs. It is generally safe to continue targeted therapy or immunotherapy for Grades 1–2 reactions (**Table 1**) but recommend holding Grade 3 and beyond. Although patients and providers may be concerned by the appearance of a rash covering up to 30% BSA, we recommend using CTCAE guidelines to determine severity and appropriate treatment and not halting immunotherapy immediately. For all patients starting a new therapy, we recommend providers do a thorough preliminary skin exam and explain common dermatologic toxicities along with warning symptoms such as significant BSA involvement, severe pain, or inability to perform activities of daily living. Proactive interventions should also be clearly communicated to patients prior to therapy. Recommended discussion points include education on avoiding tight clothing, exposure to sunlight without photoprotection, products that cause dry skin, depilatory wax and plucking, and alcohol-based cleansers and cosmetics (15).

## Conclusion

The treatment of H&N cancers has been revolutionized by the development of targeted therapies and immunotherapies. Patients with cancers that were unresponsive to traditional chemotherapies now have more targeted treatment options which overall have a better side effect profile; however, patients may be more prone to certain dermatologic toxicities. Cetuximab, trastuzumab, and afatinib commonly lead to papulopustular eruptions, xerosis, and hair and nail changes. Pembrolizumab and nivolumab can have a wide range of dermatologic findings including pruritus, morbiliform eruptions, vitiligo, bullous pemphigoid, and more. Lenvatinib, a VEGF inhibitor, may lead to stomatitis, PPES, and other side effects commonly seen in this drug class. Finally, larotrectinib and entrectinib tend to have limited non-specific cutaneous adverse events but as other immunotherapies, patients on these treatments should be closely monitored. Appropriate characterization and staging of these dermatologic toxicities can lead to better outcomes and improved patient quality of life by allowing patients to stay on the targeted therapy if the dermatologic toxicities are adequately managed. Most papulopustular and morbilliform eruptions up to Grade 3 may be treated with supportive care and the targeted therapy can be safely continued; however, more severe reactions may require temporary or permanent cessation of therapy. We recommend conducting a thorough skin exam and providing patient education on common cutaneous toxicities prior to initiation of any new therapy so that patients know what to monitor for and report to their clinicians. Additionally, patients may be counseled on proactive measures such as wearing loose clothing, applying moisturizing emollients, and using sunscreen to optimize skin health. In conclusion, patients on targeted therapy and immunotherapy may experience unique dermatologic toxicities that can be appropriately managed in order to continue their life-saving therapies.

## Author Contributions

CA, YK, ME, and FA contributed to writing the manuscript. ME and FA edited the entirety of the manuscript. All authors contributed to the article and approved the submitted version.

## Conflict of Interest

The authors declare that the research was conducted in the absence of any commercial or financial relationships that could be construed as a potential conflict of interest.
